# Impacts of artificial light at night in marine ecosystems—A review

**DOI:** 10.1111/gcb.16264

**Published:** 2022-06-14

**Authors:** Laura F. B. Marangoni, Thomas Davies, Tim Smyth, Airam Rodríguez, Mark Hamann, Cristian Duarte, Kellie Pendoley, Jørgen Berge, Elena Maggi, Oren Levy

**Affiliations:** ^1^ Smithsonian Tropical Research Institute Smithsonian Institution Ciudad de Panamá Panamá; ^2^ School of Biological and Marine Sciences University of Plymouth Plymouth Devon UK; ^3^ Plymouth Marine Laboratory, Prospect Place Plymouth Devon UK; ^4^ Grupo de Ornitología e Historia Natural de las islas Canarias, GOHNIC Buenavista del Norte Canary Islands Spain; ^5^ Terrestrial Ecology Group, Department of Ecology Universidad Autónoma de Madrid Madrid Spain; ^6^ Centro de Investigación en Biodiversidad y Cambio Global (CIBC‐UAM) Universidad Autónoma de Madrid Madrid Spain; ^7^ College of Science and Engineering, Marine Biology James Cook University Townsville Australia; ^8^ Departamento de Ecología y Biodiversidad, Facultad de Ciencias de la Vida Universidad Andres Bello Santiago Chile; ^9^ Pendoley Environmental Pty Ltd Booragoon Australia; ^10^ Department for Arctic and Marine Biology, Faculty for Biosciences, Fisheries and Economics UiT The Arctic University of Norway Tromsø Norway; ^11^ University Centre in Svalbard Longyearbyen Norway; ^12^ Department of Biology and Technology, Centre of Autonomous Marine Operations and Systems Norwegian University of Science and Technology Trondheim Norway; ^13^ Dip. di Biologia, CoNISMa Università di Pisa Pisa Italy; ^14^ Mina and Everard Goodman Faculty of Life Sciences Bar‐Ilan University Ramat Gan Israel; ^15^ The Interuniversity Institute for Marine Sciences, The H. Steinitz Marine Biology Laboratory Eilat Israel

**Keywords:** artificial light at night (ALAN), conservation guidelines, coral reefs, marine ecosystem, pelagic organisms, rocky intertidal shores, sandy beach, seabirds, sea‐turtles

## Abstract

The globally widespread adoption of Artificial Light at Night (ALAN) began in the mid‐20th century. Yet, it is only in the last decade that a renewed research focus has emerged into its impacts on ecological and biological processes in the marine environment that are guided by natural intensities, moon phase, natural light and dark cycles and daily light spectra alterations. The field has diversified rapidly from one restricted to impacts on a handful of vertebrates, to one in which impacts have been quantified across a broad array of marine and coastal habitats and species. Here, we review the current understanding of ALAN impacts in diverse marine ecosystems. The review presents the current state of knowledge across key marine and coastal ecosystems (sandy and rocky shores, coral reefs and pelagic) and taxa (birds and sea turtles), introducing how ALAN can mask seabird and sea turtle navigation, cause changes in animals predation patterns and failure of coral spawning synchronization, as well as inhibition of zooplankton Diel Vertical Migration. Mitigation measures are recommended, however, while strategies for mitigation were easily identified, barriers to implementation are poorly understood. Finally, we point out knowledge gaps that if addressed would aid in the prediction and mitigation of ALAN impacts in the marine realm.

## Graphical abstract

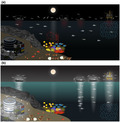

## 1 INTRODUCTION

Artificial Light at Night (ALAN) is a widespread, pervasive, and expanding form of pollution (Gaston et al., 2021) that has come to be recognized as a major 21st century global change issue (Davies & Smyth, 2018). Its impacts span the biological hierarchy ranging from those on organism physiology through to changes in the composition of ecological communities (Sanders et al., 2021). It is now broadly accepted that ALAN has been reshaping nature for more than a century.

**FIGURE 1:**
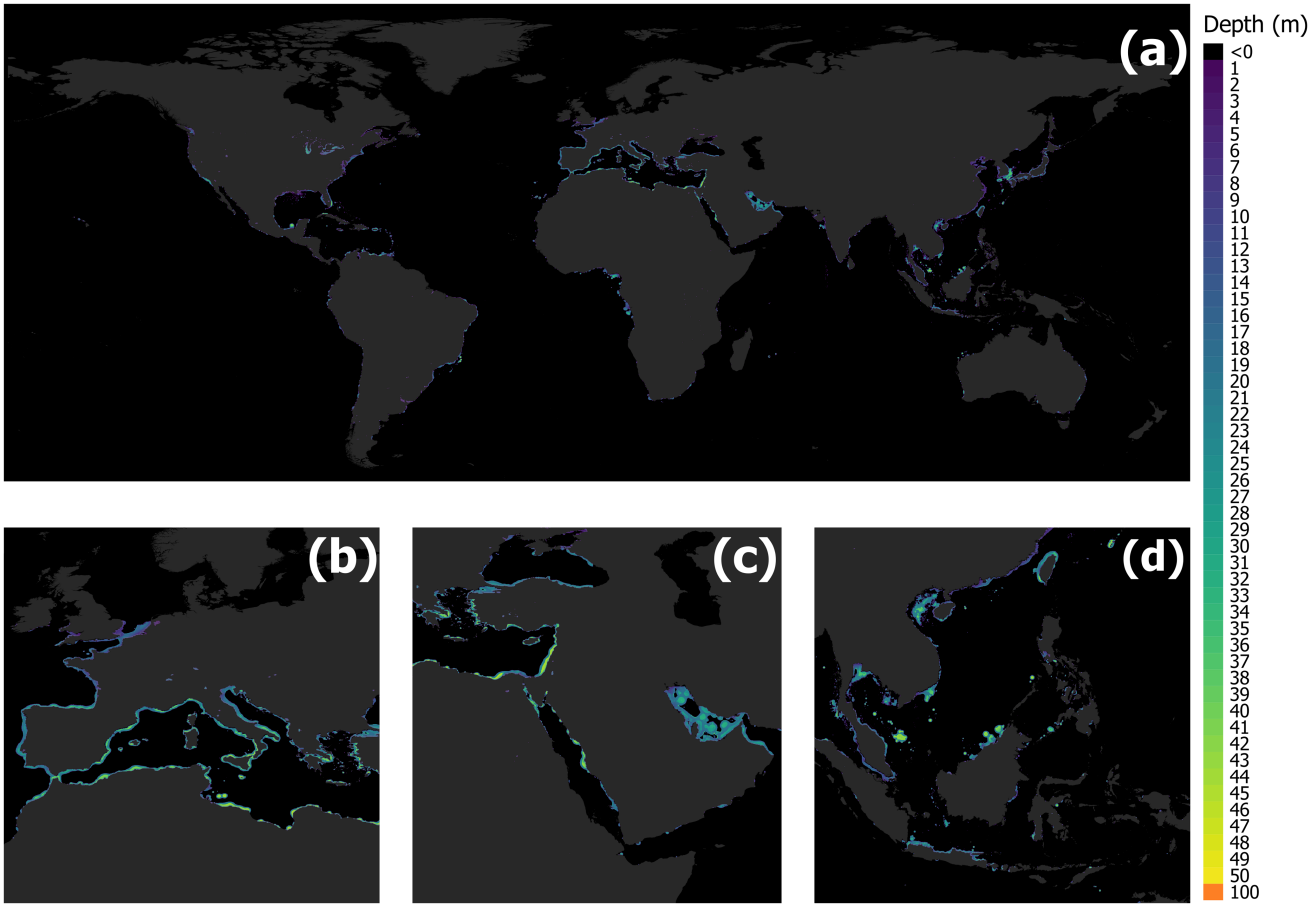
The depth of biologically important artificial light at night (ALAN) (*Zc*): around the world's coastlines (a); in the Mediterranean and Northeast Atlantic (b); in the Black Sea, the Red Sea and the Persian Gulf (c); and in the Gulf of Thailand, Andaman Sea, South China Sea and the Java Sea (d). The legend inset details the depths (m) to which biologically important ALAN penetrates the sea. The data is derived from the relationship between The New World Atlas of Artificial Night Sky Brightness (Falchi et al., 2016) and sea surface irradiances (Davies et al., 2020), coupled with the monthly climatologies of globally inherent optical water properties and validated against in situ data collected from the Persian Gulf (Tamir et al., 2017). “Biologically important ALAN” is defined as the minimum irradiances of white light that elicit diel vertical migration in female *Calanus* copepods (Båtnes et al., 2013) [see Smyth et al., 2021 for further details]. Maps are representative of average ALAN penetration over a typical year. Full dataset is available to download from https://doi.pangaea.de/10.1594/PANGAEA.929749.

Although research into the prevalence and impacts of ALAN in marine ecosystems has somewhat lagged behind terrestrial, the last 5 years have seen a dramatic advance in our understanding. We now know that at least 22% of coastal regions are exposed to ALAN (Davies et al., 2014), and the light from cities is sufficient to elicit biological responses in animals on the seafloor in adjacent habitats (Ayalon, Rosenberg, et al., 2021; Davies et al., 2020). 1.9 million km^2^ of the world's coastal seas are exposed to ALAN at 1 m depth, 1.6 million km^2^ at 10 m depth, and 840,000 km^2^ at 20 m depth (Figure 1a) (Smyth et al., 2021). The most exposed regions include the Mediterranean (Figure 1b), the Red Sea and Persian Gulf (Figure 1c), and the seas of South‐East Asia (Figure 1d). ALAN is even prevalent across those areas of the ocean most valued by humanity, with 20% of the world's contiguous Marine Protected Areas exposed across 100% of their range (Davies et al., 2016).

**FIGURE 2:**
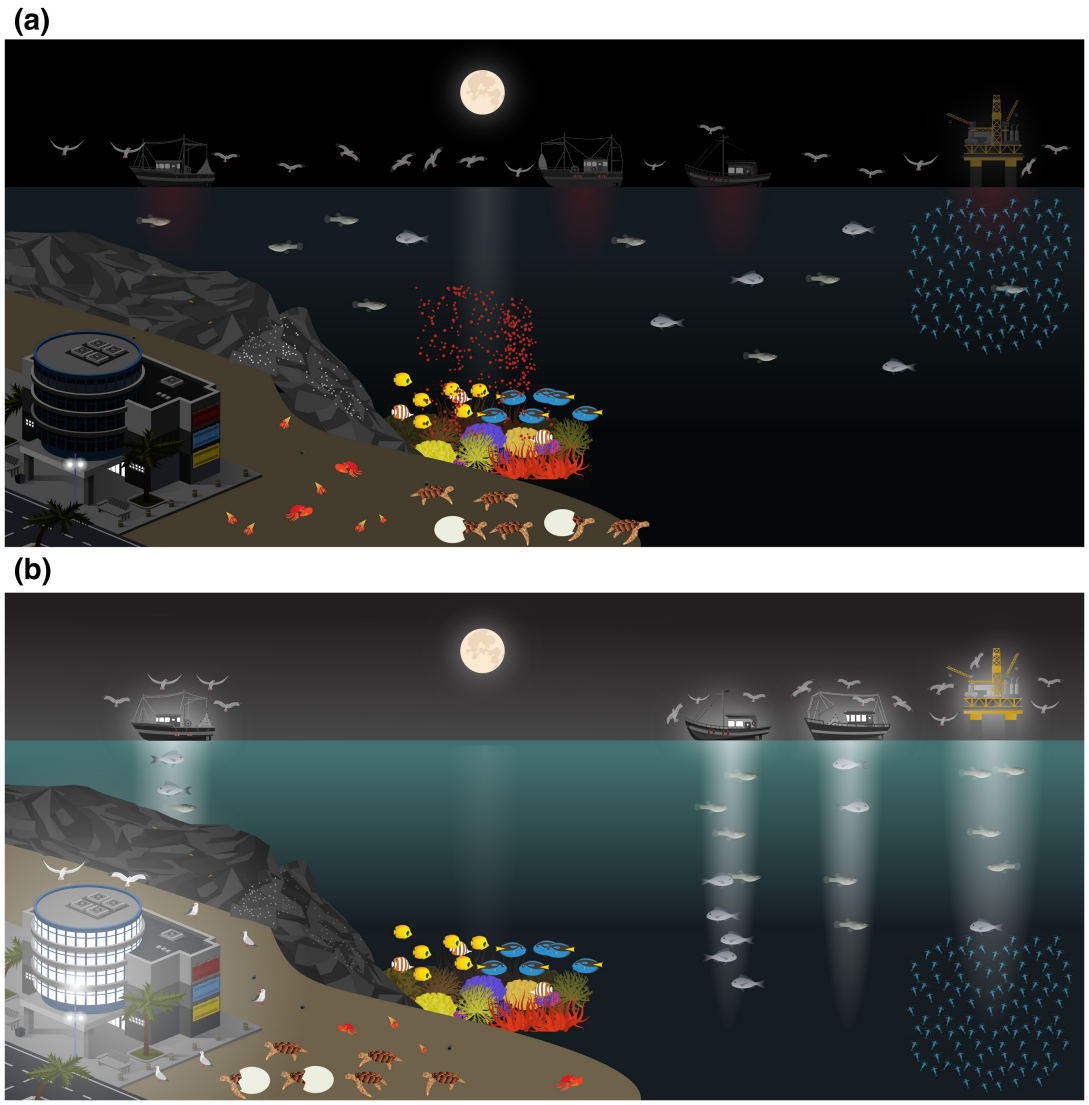
(a) Different marine environments not affected by Artificial Light Pollution at Night (ALAN), and (b) marine environments under the potential impacts of ALAN: (i) Sandy beaches effects on invertebrate species day‐night activity rhythms and biodiversity, effects in the on‐beach orientation of adults and hatchling turtles, and seabirds fledgling grounded by ALAN; (ii) Rocky intertidal shores – influence in metabolic activity/behavior of primary producers, sessile and mobile animals; (iii) Shallow water coral reefs – effects on gametogenesis and the synchronization of gamete release in prominent coral species, and negative impacts over fish reproduction, (iv) Pelagic environment – inhibition of vertically migrating zooplankton, and disorientation and mortality of seabirds.

The potential for ALAN to impact the wide array of organisms, processes, and habitats in the sea for which light cycles are critical had remained largely unexplored until recently (Davies et al., 2014; Longcore & Rich, 2004). These include: diel vertical migrations (Berge et al., 2020)—the largest migration of biomass on the planet (Hayes, 2003); coral spawning (Ayalon, Rosenberg, et al., 2021), and symbiosis (Ayalon, Benichou, et al., 2021)—which are key for the maintenance of coral reefs; consumer‐resource interactions (Bolton et al., 2017; Maggi, Bongiorni, et al., 2020; Underwood et al., 2017) that are known to drive top down structuring of marine ecosystems (Paine, 1966); migrations and orientation of marine organisms—critical for their survival (Navarro‐Barranco & Hughes, 2015; Torres et al., 2020); and the recruitment of sessile invertebrate larvae into marine habitats (Davies et al., 2015; Lynn, Quintanilla‐Ahumada, et al., 2021), (Figure 2). All these processes are fundamental to the health of marine ecosystems, and all are known to depend on the cycles, spectra or intensity of sun or moonlight.

Here, we bring together recognized experts in marine ALAN across habitats and taxonomic groups to review these recent advances, with the aim of providing a gateway to research in the field. First, we review progress in sandy beaches, rocky intertidal shores, shallow water coral reefs, and pelagic environments (Figure 2). Second, we evaluate the current state of litigation and management options available to conservation practitioners. Finally, we identify key knowledge gaps and highlight key questions for future research.

## 2 BIOLOGICAL EFFECTS OF ALAN IN THE MARINE BIOTA

In this section we have compiled the most relevant information obtained on key species belonging to different marine habitats across sandy beaches (including sea turtles), rocky intertidal shores, shallow water coral reefs, and pelagic environments. A special section was created for seabirds due to their high mobility and, therefore, presence in different marine realms. Despite the research gaps and limitations—to name a few, for example, lack of multistressor experiments including ALAN as a factor, and difficulties to acquire data to define more precisely ecological relevant light intensities to be tested (see Box 1) (Aulsebrook et al., 2022)—ALAN can be recognized as major sensory pollutant of concern due it is obvious and widespread effects on pathways associated with natural circadian regulations in the marine biota. It is also important to note that experimental approaches using high ALAN levels (not considered environmental realistic) in many of the short‐term experiments presented here consist in an important step to understanding the mechanisms and long‐term effects of the chronic disturbance caused by ALAN.

BOX 1 **Challenges in measuring artificial light in biological studies**

Measuring light for ecological studies is still undefined and is poorly understood by biologists who have used a wide range of techniques and instruments to measure light at scales ranging from a few centimeters to tens of kilometers. The wide range of instruments employed typically use different measurement systems and units, which means study results cannot be confidently compared. There is currently no globally recognized standard method, or agreed unit of measurement, for monitoring biologically active light (Barentine, 2019).

The figure below demonstrates this. Commercial lux meters only quantify light that is within the CIE curve (area under grey dashed line) and exclude light wavelengths that fall outside of the CIE curve.

Credit: Kellie Pendoley.

Visible light can be described by two physical parameters: wavelength and intensity. The relative distribution and weighting of different wavelengths in emitted light determines its color; however, how this light is perceived by the observer is also a function of the physiology of the receiving detector (e.g., eye). The intensity of light reaching a detector is a function of distance from the light source as light waves spread out from the light source the number of waves falling on a defined area decreases proportional to the distance travelled. Light is measured either radiometrically or photometrically. Radiometry is the measurement of wavelengths across the entire electromagnetic spectrum. In biological applications this is typically restricted to the ultraviolet, visible, and infrared region of the spectrum between 350 and 800 nm and is measured in watts per meter squared. Most commercial light measurement equipment records photometric light which is visible light wavelengths weighted specifically to the sensitivity of the human eye (CIE, 2005) and is reported in units of lumens per meter squared or Lux. Photometric detectors have reduced sensitivity to wavelengths below 450 nm (blue light) or above 650 nm (red light). Consequently, photometric instruments commonly used to quantify light in biological studies, such as lux meters, do not account for the blue light that is most visible to biological receptors.
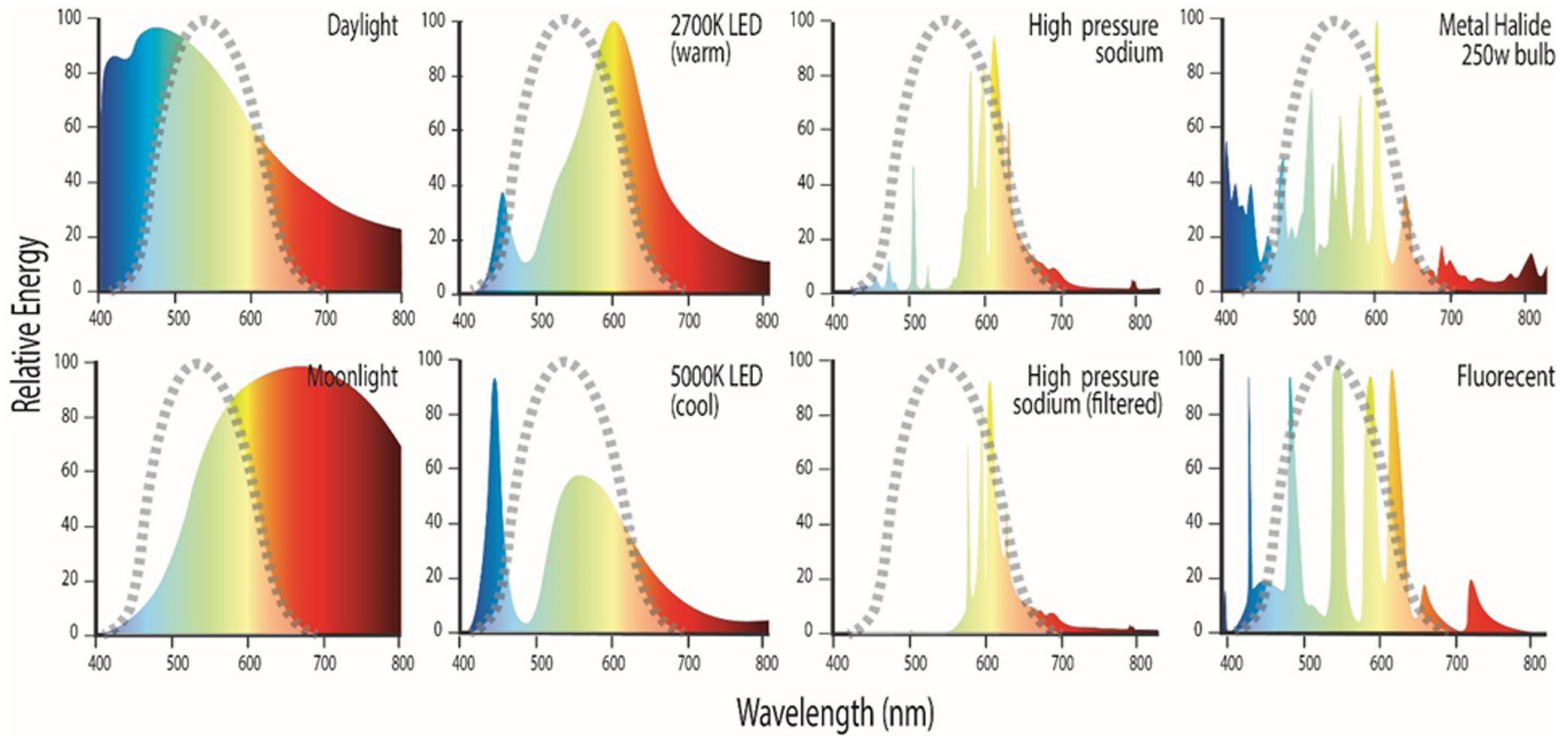

Lux meters are further limited in that they were designed for use in measuring light in buildings, have poor sensitivity to low light levels and cannot detect sky glow or dim light in a field setting. Finally, they do not provide any spectral information, i.e., the relative distribution of light wavelengths, in the light source.

The limitations and challenges around measuring ecological light on scales ranging from landscape to small scale is discussed in more detail in Commonwealth (2020); Longcore et al. (2020), Hänel et al. (2018), Jechow and Hölker (2019) and Jechow et al. (2019).

### 2.1 Sandy beach ecosystems

**FIGURE 3:**
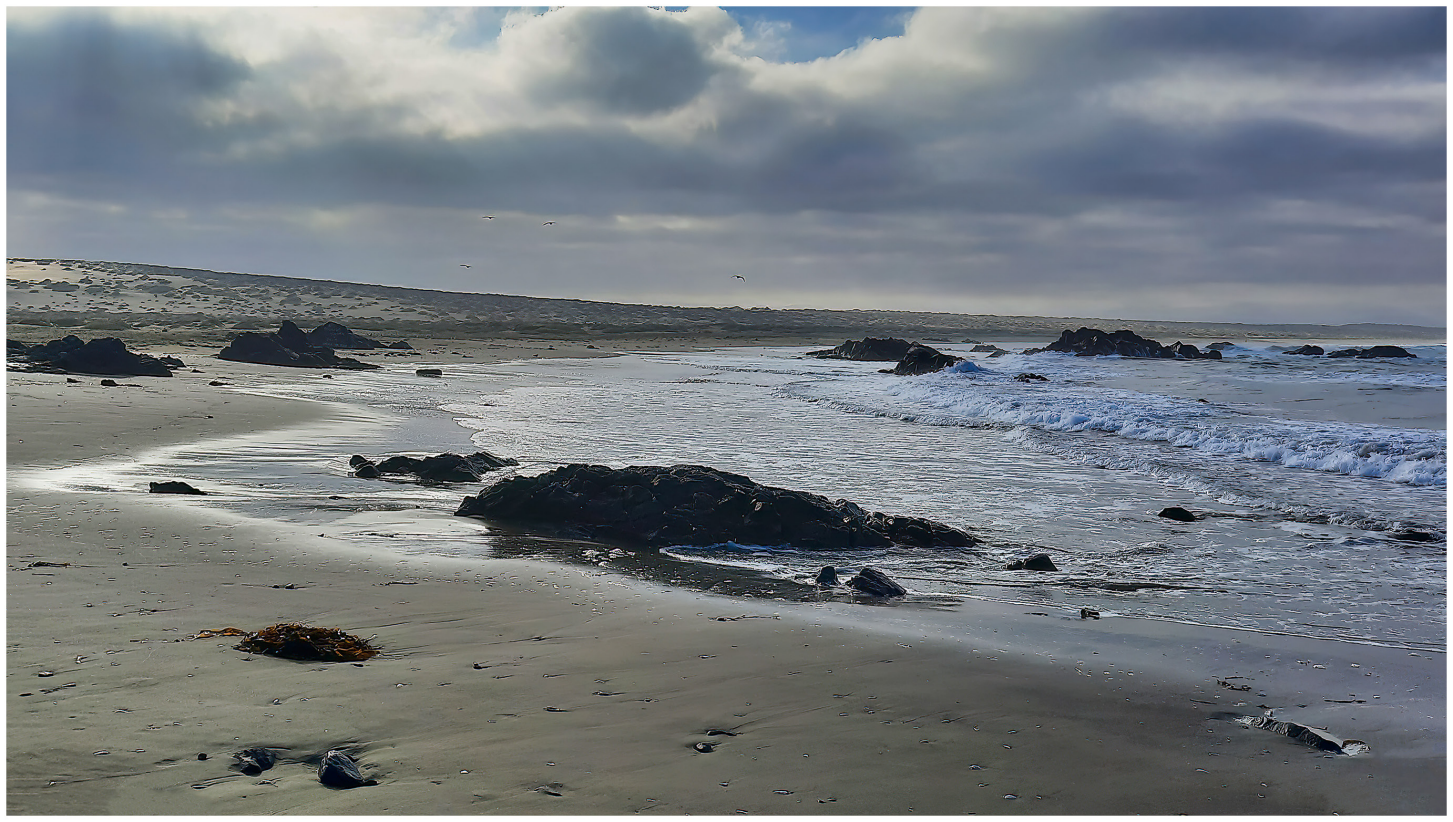
Los Choros Sandy beach, coast of Coquimbo, North of Chile (Credit; Josué Navarrete).

Many sandy beach species are known for their day/night activity rhythms controlled by natural light cycles. Consequently, the dramatic ongoing expansion of ALAN in these ecosystems is expected to exert a significant effect on sandy beach biodiversity (Figure 3).

Exposed sandy beach ecosystems represent over 80% of the ice‐free coastline (Bascom, 1980). These ecosystems are considered highly valuable from an ecologic, economic and cultural points of view (King & Symes, 2004; McLachlan et al., 2013; Pendleton et al., 2006). However, the growing urbanization and the increase of human population near sandy shores has become a threat to these ecosystems (Jaramillo et al., 2021). One of the most important environmental stressors associated with the urbanization of sandy beaches is ALAN (Gonzalez et al., 2014; Schlacher et al., 2016).

Sandy beaches support an abundant and diverse fauna, being crustaceans (mainly talitrid amphipods, cirolanid and oniscoid isopods and hippid crabs), polychaetes and bivalves the main taxonomic groups (Brown & McLachlan, 1990; Jaramillo et al., 2003). Several sandy beach species such as amphipods, isopods, and insects are known for their dynamic day/night activity rhythms (Fallaci et al., 1999, 2002; Jaramillo et al., 2003; Kennedy et al., 2000), which are controlled by natural geophysical cycles of day and night, and moonlight (Jaramillo et al., 2003; Meschini et al., 2008; Ugolini et al., 2003). For example, these invertebrates rely on darkness to use natural light signals and return to their burrows after down shore migrations (Fallaci et al., 2002). Consequently, the ongoing expansion of ALAN in these ecosystems is expected to exert both a significant and negative effect on sandy beach biodiversity (e.g., Duarte et al., 2019; Luarte et al., 2016). However, despite growing concerns on the impacts of ALAN, studies evaluating its effects on sandy beach organisms, other than sea turtles (e.g., Rivas et al., 2015; Dimitriadis et al., 2018, see specific section 2.5. for this group), remain very scarce (e.g., Lynn, Flynn, et al., 2021; Quintanilla‐Ahumada et al., 2021). The studies carried out so far have evaluated the effects of ALAN on locomotor activity, feeding rates, absorption efficiency and growth rate, physiology, distribution, and abundance of different species of invertebrates (see below).

One of the first studies evaluating the ALAN effects on locomotor activity of sandy beach animals was conducted by Bregazzi and Naylor (1972). Those authors showed that in laboratory conditions ALAN (~ 200 lux) almost entirely inhibited the locomotor activity of the talitrid amphipod *Talitrus saltator*. Interestingly, these individuals recovered their locomotor activity pattern immediately after the light was removed. Similar results were registered by Luarte et al. (2016), who using laboratory experiments showed that the locomotor activity of the talitrid amphipod *Orchestoidea tuberculata* was significantly reduced in the presence of ALAN (60 lux). Similarly, Duarte et al. (2019) found that in laboratory conditions (120 lux), ALAN reduced the locomotor activity of the oniscoid isopod *Tylus spinulosus*. More recently, Lynn et al. (2021,b) also found that ALAN (80 lux) disrupted the locomotor activity of the talitrid amphipod *Americorchestia longicornis*. Consistent with the results reported by Bregazzi and Naylor (1972), *A. longicornis* was found to recover its natural rhythm of activity shortly after ALAN was removed from the system (Lynn, Flynn, et al., 2021). By contrast, Fanini et al. (2016) found that the locomotor activity of the amphipod *Platorchestia smithi* was similar in a beach exposed to ALAN with respect to another that was not exposed. However, this last study was correlational and should be considered with caution. More recently 0.2 lux white lighting lower in brightness than a full moon (equivalent to artificial sky glow) has been demonstrated to reduce locomotor activity and disorientate the migration behavior of *Talitrus saltator* (Torres et al., 2020).

Only a scarce number of studies have evaluated ALAN effects on aspects such as feeding rates, absorption efficiency and growth rates (Luarte et al., 2016; Lynn, Flynn, et al., 2021; Quintanilla‐Ahumada et al., 2022). The presence of ALAN reduced the consumption rate, absorption efficiency and growth rate in the amphipods *O. tuberculata* (Luarte et al., 2016) and *A. longicornis* (Lynn, Flynn, et al., 2021), meanwhile ALAN (from 0 to 100 lx) did not affect *T. spinulosus*'s growth rate but increased its absorption efficiency (Quintanilla‐Ahumada et al., 2022). The absorption efficiency results should be considered with precaution, because of methodological restrictions, as the animals had to be maintained without sand, which, eventually, could modify the ALAN effects on this biological trait). The RNA:DNA ratio is a relatively new indicator of the physiological or nutritional condition of organisms (Buckley et al., 1999; Chícharo & Chícharo, 2008). Recent studies by Quintanilla‐Ahumada et al. (2021, 2022), used this molecular tool to evaluate ALAN effects in sandy beach ecosystems, found that RNA:DNA ratio in the insect *Phalerisida maculata* and in the isopod *T. spinulosus,* declined in the presence of ALAN, indicating detrimental physiological effects. ALAN also shows important effects on the abundance and distribution of sandy beach organisms. Gonzalez et al. (2014) applied correlative analyses and found that the abundance of the insect *P. maculata* was negatively related with the night sky quality (an indirect indicator of ALAN). Duarte et al. (2019) registered that the distribution of *T. spinulosus* was modified by ALAN (120 lux and less), with individuals avoiding the lit areas and therefore restricting their habitat availability. Importantly, ALAN effects decreased with increasing distance from the light sources (Duarte et al., 2019). Despite the work conducted this far, direct evidence of ALAN effects on sandy beach organisms remains limited only to studies focusing on this stressor upon single species (e.g., Duarte et al., 2019; Luarte et al., 2016).

It is important to note that the light intensities used in some of these studies were those recorded under the light source (mainly in Chilean sandy beaches) in the promenade area, located very close to the beach. Such light intensities could be higher than those occurring in the intertidal area, mainly in the middle and lower intertidal zone. Therefore, future studies should consider using the light intensity directly recorded at the intertidal zone. However, at least for the Chilean coast, in extreme heavy polluted beaches, light intensity values in the intertidal zone can be as high or higher than those used in these studies, mainly in the upper intertidal zone (Duarte unpublished data). Another important consideration should be to expand ALAN studies to beaches located in different geographic areas, for example tropical ones, which with the exception of sea turtle species, have not received special attention regarding to ALAN.

#### 2.1.1 Sea‐Turtles

**FIGURE 4:**
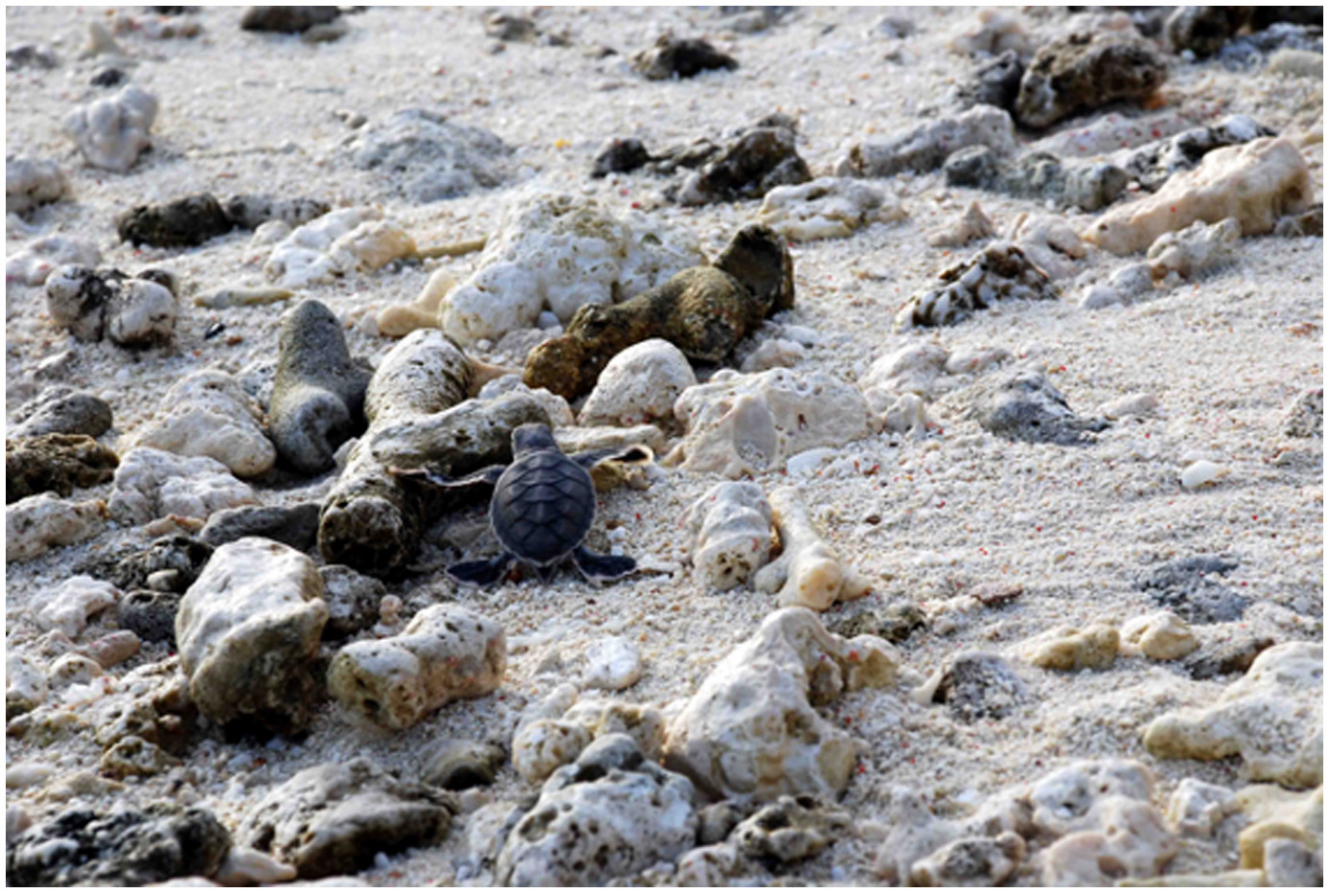
Sea turtles hatching in Heron Island, Australia (Credit; Levy, O).

It is well established that ALAN, even at low levels, is a threat influencing all seven species of marine turtle, primarily as hatchlings or nesting adult females (Salmon, 2003; Witherington & Bjorndal, 1991a, 1991b) (Figure 4). Artificial light pollution is known to influence (i) the on‐beach orientation and nest site selection of adult female turtles, (ii) the on‐beach orientation and sea finding behavior of hatchling turtles, and (iii) the at‐sea dispersal of hatchling turtles. The degree to which species, or populations are exposed to artificial light pollution, and thus its influence as a threatening process, varies across the world with populations nesting at sites closer to areas of urban or industrial development being more exposed (e.g., Colman et al., 2020; Kamrowski et al., 2012, 2014; Shimada et al., 2021).

Artificial light primarily impacts marine turtles during nesting or hatchling life stages, and consequently it is predominantly linked to early life stage mortality. Thus, it could, along with other threats, cause gradual decline in the reproductive output of a nesting area. However, while there are several studies examining beach specific exposure, or short‐term (season) impacts, quantifying the long‐term impacts of artificial light pollution‐caused mortality are difficult because marine turtles can take decades to reach maturity, and impacts of light pollution contribute to other pressures over turtles' lifetimes to influence population viability. Hence, understanding the degree to which nesting sites for each population of turtles are exposed to artificial light pollution is important, so that site‐specific interventions can be implemented.

Typically, female turtles lay their clutches above the high‐water mark, and often in the primary dune systems or high up on the beach. Hatchlings generally emerge from nests at night and orient themselves from the nest site to the ocean, ideally as fast as possible (Wyneken & Salmon, 1992). After emerging they generally show a preference of moving towards horizons which are low and bright, and moving away from horizons, which are dark and elevated (e.g., Limpus & Kamrowski, 2013; Lucas et al., 1992; Pendoley & Kamrowski, 2015; Salmon et al., 1995) and using these cues they can navigate across the beach to the water. Exposure to coastal light pollution disrupts the natural orientation cues and leads to the disorientation and misorientation of hatchlings because lights obscure the natural horizons (Witherington & Bjorndal, 1991a, 1991b).

Hatchling sea finding ability is influenced by both the wavelength and intensity of artificial light (Cruz et al., 2018; Witherington & Bjorndal, 1991b). Such ability is significantly compromised by exposure to shorter wavelength lights, even at lower intensity (Celano et al., 2018; Salmon, 2003). Importantly, it is becoming clear that the thresholds of concern for both wavelength and intensity of artificial light are likely to vary within and among species (Fritsches, 2012). The impacts of exposure can be influenced by the presence or absence of other natural (dune height and structure, vegetation) or unnatural cues (presence of buildings or artificial structures) (Kamrowski et al., 2015; Salmon, 2003), highlighting the need for site‐specific research on orientation thresholds and light‐reduction interventions.

Once at sea, hatchling turtles will swim actively for around 24 to 48 h (Wyneken & Salmon, 1992). This period, known as the swim frenzy, enables hatchlings to move quickly from nearshore to offshore waters (Wyneken & Salmon, 1992). During the swim frenzy the hatchlings are using multiple cues to enable their directional swimming – these include swimming towards the low, light, horizon, and swimming perpendicular to wave fronts (Lohmann et al., 2017; Salmon & Wyneken, 1987; Wilson et al., 2021). According to Salmon and Wyneken (1987) light cues are important for at sea dispersal, however, there is likely to be a distance offshore where the cue is either not available or not used. Although this distance is currently unknown, there is a growing empirical basis demonstrating that offshore dispersal for marine turtle hatchlings is compromised by light pollution originating from land‐based or marine structures, such as infrastructure like jetties (Cruz et al., 2018; Truscott et al., 2017; Wilson et al., 2018). Continuing to advance knowledge on how the at sea dispersal is influenced by artificial lights from shore, or offshore infrastructure is a key avenue for further research as human developments expand along the coasts.

For nesting turtles there has been substantial research on factors that influence nest site selection (i.e., the placement of clutches on a beach). Factors including distance from vegetation (most nests being laid closer to the vegetation line—Hays et al., 1995; Kelly et al., 2017), elevation and beach slope (Patrício et al., 2018; Wood & Bjorndal, 2000) and exposure to artificial light pollution (Salmon, 2003; Windle et al., 2018) have all been associated with nest site selection. There is also variation among species and region as to the relative importance of each. However, less research has been conducted to examine the influence of light pollution on nest site selection by nesting turtles. Among the studies, Salmon (2003) used data from a main nesting area in Florida to test nest site selection in relation to the degree females were exposed to artificial light spill onto beaches; and Windle et al. (2018) used a combination of light pollution data and turtle density data to examine the influence of artificial light pollution on nest site selection. Both studies concluded that turtles use darker beaches and select darker sections of beaches.

### 2.2 Rocky intertidal shores

**FIGURE 5:**
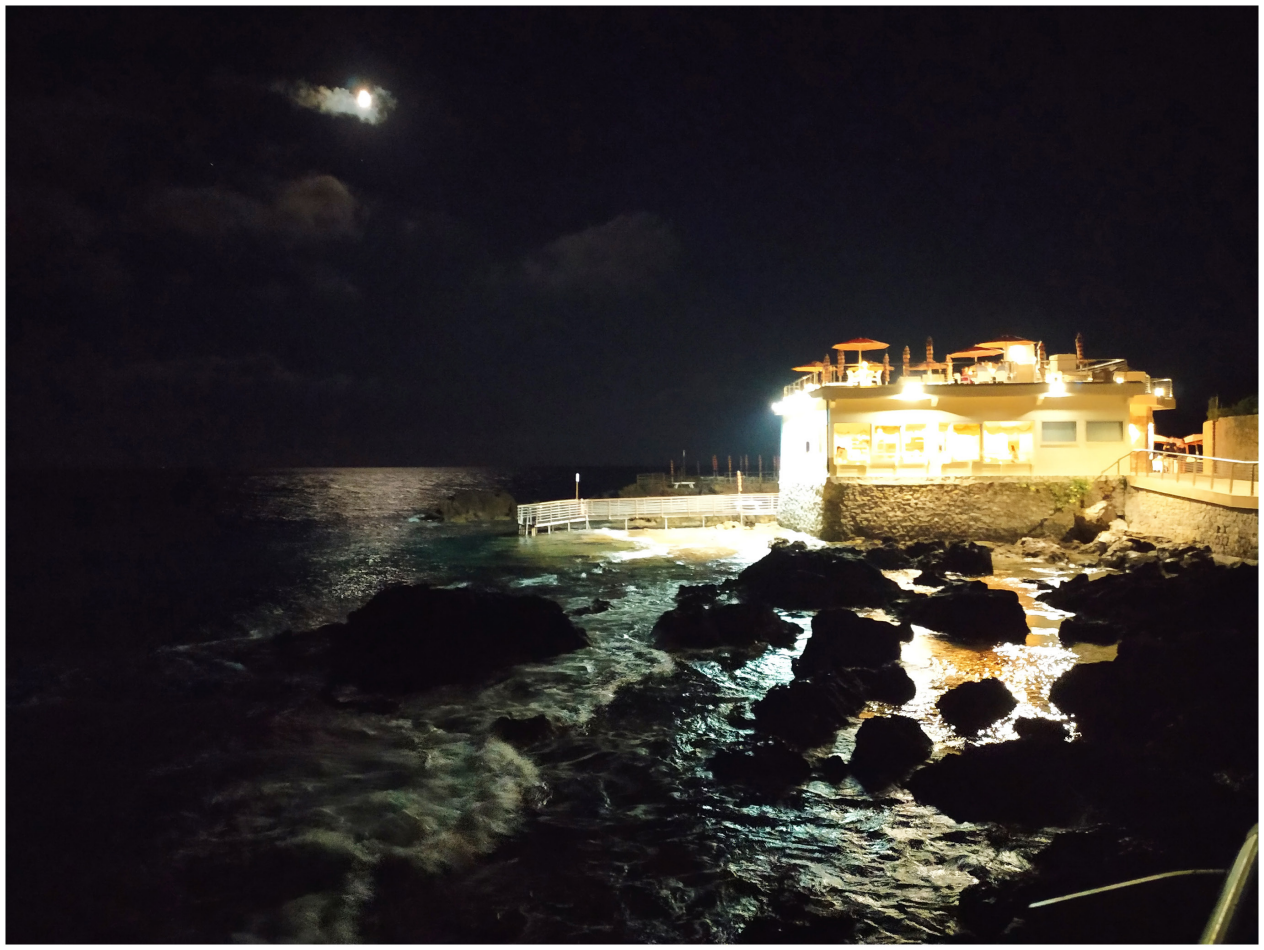
Intertidal rocky shore in an urban area, Italy (Credit; Elena Maggi).

On a global scale, rocky intertidal shores are inhabited by diverse assemblages largely influenced by light for metabolic activities and behaviors at different life stages. It is reasonable to expect that the presence of ALAN may influence microphytobenthic and macroalgal primary producers, as well as sessile and mobile animals (Figure 5).

Intertidal habitats represent a thin line demarcating the boundary between land and marine masses. Among intertidal environments, rocky shores are characterized by large variations in abiotic conditions, including strong gradients in wave exposure, temperature, and desiccation. Despite the importance of such extreme factors, biotic interactions have shown strong determinants of distribution patterns and abundance of organisms. These features have historically made rocky shore intertidal habitats a natural laboratory to explore physiological and ecological processes and mechanisms, which have been responsible for key conceptual advancements on ecosystem functioning (Menge & Branch, 2001). ALAN, however, represents a quite novel and peculiar source of disturbance, whose effects are hardly predictable from knowledge on other stressors. In fact, it is expected to act on pathways associated with natural circadian regulations, which are related to different light optima and sensitivities among species and life stages (Davies & Smyth, 2018). Although to date the literature on effects of ALAN on intertidal rocky shores is not particularly numerous, effects at the scale of both the individual and the ecosystem have already been highlighted.

Intertidal rocky shores host a diverse array of organisms, which can be primarily categorized into sessile and vagile. Sessile species are those most diverse in terms of both size, taxonomy and trophic category. A large percentage of these species belongs to microbial biofilm. Despite low visibility, its autotrophic component microphytobenthos, MPB) represents one of the main groups of primary producers in intertidal habitats and, in association with heterotrophic microorganisms, a source of food for a plethora of grazers species (Jenkins & Hartnoll, 2001; Nagarkar et al., 2004). Seaweeds are the macroscopic primary producers on intertidal rocky shores, sometimes present as dense macroalgal beds able to modify the abiotic conditions on the shore through their canopies. Sessile species also include a variety of animals, such as bivalves, barnacles, ascidians, bryozoans, hydrozoans, gastropods and polychaetes, among others. Mobile animals are abundant and diversified as well; these include either herbivores, carnivores and omnivores almost freely living on the different vertical portions of the intertidal habitats (e.g., limpets, gastropods, crabs, sea stars, sea urchins, small fish), or smaller species more strictly associated with larger ones, such amphipods, isopods and crustaceans.

In the microtidal Mediterranean system, where intertidal biofilm is dominated by bacteria, the presence of lit areas at night at intensities typically found along the coast (27 lux) was able to increase the biomass of MPB (here dominated by cyanobacteria) and its photosynthetic efficiency (Maggi & Benedetti‐Cecchi, 2018). Subsequent studies indicated that ALAN effect was likely mediated by temporal changes in composition of mature assemblages (Maggi, Bertocci, & Benedetti‐Cecchi, 2020; Maggi, Bongiorni, et al., 2020) and possibly related to different light optima among (groups of) species. Potential consequences of effects on autotrophic microorganisms are not limited to net oxygen emissions and indirect impacts on their consumers. In fact, the composition of microbial biofilm plays a key role in the settlement of larval stages of invertebrates and spores (Keough & Raimondi, 1995; Qian et al., 2007). Indeed, the first study investigating the role of ALAN (either 30 or 19 lux) for intertidal organisms focused on sessile larval stages of animals, revealing variable effects on settlement rates on PVC panels attached on wooden floating rafts in the UK (Davies et al., 2015). Results showed that 39% of analyzed taxa were influenced by ALAN, either positively or negatively; it was not surprising, given the importance of natural light as a cue for guiding larval recruitment and later survival (Mundy & Babcock, 1998; Thorson, 1964). More recently, negative effects of ALAN on late settlers of barnacles have been documented both along natural shores in Chile (97‐11 lux) (*Notochthamalus scabrosus* and *Jehlius cirratus*; Manríquez, Jara, et al., 2021; Manríquez, Quijon, et al., 2021) and in the North Atlantic on man‐made structures (212‐11 lux) (*Semibalanus balanoides*; Lynn, Flynn, et al., 2021); in these studies, lack of effects on early settlers suggested that, in presence of lit nights, metamorphosis was delayed or early mortality was increased in comparison to natural dark conditions.

As for sessile intertidal species, a big knowledge gap on effects on autotrophs still exists, with a complete lack of studies on macroalgal species. In this case, it is worth mentioning that ALAN effects could also influence non‐trophic interactions mediated by algal canopies, such as facilitative effects exerted through the reduction of artificial light intensities for understory assemblages (Bruno & Bertness, 2001). In addition to sessile species or life stages, intertidal rocky reefs are inhabited by mobile individuals. Mobility is a great advantage in a habitat characterized by high abiotic variation. For example, many organisms have evolved predominantly nocturnal behaviors to avoid energy expenditure related to thermal stress or to reduce the risk of predation linked to visual stimuli (Manríquez et al., 2009; Wells, 1980). Different organs are involved in the perception of circadian changes in light intensity and of prey or predators, from relatively simple photoreceptors capable of forming sharp images in air in gastropods (Newell, 1965), to proper eyes in fish. Along the Chilean coasts, the abalone *Concholepas concholepas*, or “Loco,” is an ecologically and economically key species. Like most mollusks, it uses both chemical and visual stimuli during sensory perception (Domenici et al., 2017; Manríquez et al., 2014). Chemoreception of odor cues through the osphradium is the main tool to monitor the presence of food items or predators; while information detected through its simple eyes modulates phototaxis behavior and locomotion, and detection of forms. In addition, intertidal populations of the “Loco” have been observed to prey mainly at night. This has led scientists to hypothesize physiological and behavioral responses to the presence of ALAN. Indeed, field monitoring and laboratory experiments have shown an increase in metabolic rate and self‐righting time in juveniles in presence of LED illumination at night (~330 lux), as well as a preference for dark areas to choose their prey (Manriquez et al., 2019). Similarly, ALAN (~100 lux) reduced feeding activity in adult individuals, which were also more likely to be in a refuge than those under control conditions (Manríquez, Jara, et al., 2021; Manríquez, Quijon, et al., 2021). These impacts have clear implications for the long‐term sustainability and productivity of a keystone intertidal species that is able (among others) to consume the dominant mussel *Perumytilus purpuratus,* and therefore enhance rocky intertidal biodiversity and functioning. These results do not, however, appear to be generalizable across rocky shore predators. The common Atlantic dog whelk *Nucella lapillus*, forages more under ALAN even in the presence of a predator cue (Underwood et al., 2017) possibly due to increased metabolic stress and the ability to visually perceive there is no predator threat.

Like some invertebrate species, many fish species have evolved endogenous clock systems which regulate tidally and circadian organized behavioral rhythms (Helfman et al., 2009). Among them is the “Baunco”, the rockfish *Girella laevifrons*, one of the most abundant fish in the littoral zone of Southeastern Pacific. A recent study showed an increase in oxygen consumption and activity of this fish under ALAN conditions (70 lux). Importantly, loss of a dark night period was able to modify or even stop the daily peak of activity of the Baunco, posing serious questions about the sustainability of intertidal fish populations in urban areas (Pulgar et al., 2019). Results are in accordance with those obtained on fish by manipulating ALAN under a wharf in Sydney Harbour (Bolton et al., 2017). The latter showed that, under ALAN, predatory behavior was dramatically greater while abundance was reduced, a similar condition observed during daylight conditions. Interestingly, authors observed a concomitant change in the structure of prey (epifaunal) communities, which suggested an indirect top‐down effect of ALAN. It is interesting to note that the presence of night lighting of artificial structures might elicit variable effects on fish depending on the positioning of the light source. Lamps positioned over the structure, for example, create an unnatural high contrast between illuminated areas and darker surroundings, these latter found to attract higher densities of bogues (*Boops boops*) possibly seeking for a refuge (Georgiadis et al., 2014; Mavraki et al., 2016). Further variability is likely related to species‐specific responses, as suggested by the almost lack of effects on juvenile bonefish (Szekeres et al., 2017) or on most fish assemblage inhabiting shallow coastal seagrass beds (Martin et al., 2021).

The mentioned studies suggest that the effect of ALAN on single organisms can easily reverberate on entire communities through trophic and non‐trophic interactions. Further, manipulative experiments in the Mediterranean rocky intertidal on biofilm assemblages and their main consumers (i.e., littorinid gastropods) highlighted quite complex and temporally variable effects. On the short term (~1 month), the increase in consumer pressure by grazers compensated for the positive effect of ALAN (27 lux) on microbial primary producers (MPB), indicating that trophic interactions can provide a stabilizing mechanism against the effects of light pollution on early colonizing autotrophs (Maggi & Benedetti‐Cecchi, 2018). On the contrary, on longer terms (~3 months), the presence of ALAN negatively affected the density of grazers. Adding to the positive effect on cyanobacteria, ALAN eventually promoted a shift in the composition of epilithic assemblages, characterized by a higher photoautotrophic diversity at the expense of heterotrophic bacteria (Maggi, Bertocci, & Benedetti‐Cecchi, 2020; Maggi, Bongiorni, et al., 2020). Although it is clear how pervasive the role of light pollution on rocky shore intertidal communities is, biological and ecological consequences on different species and communities are still largely unknown.

### 2.3 Shallow water coral reef ecosystems

**FIGURE 6:**
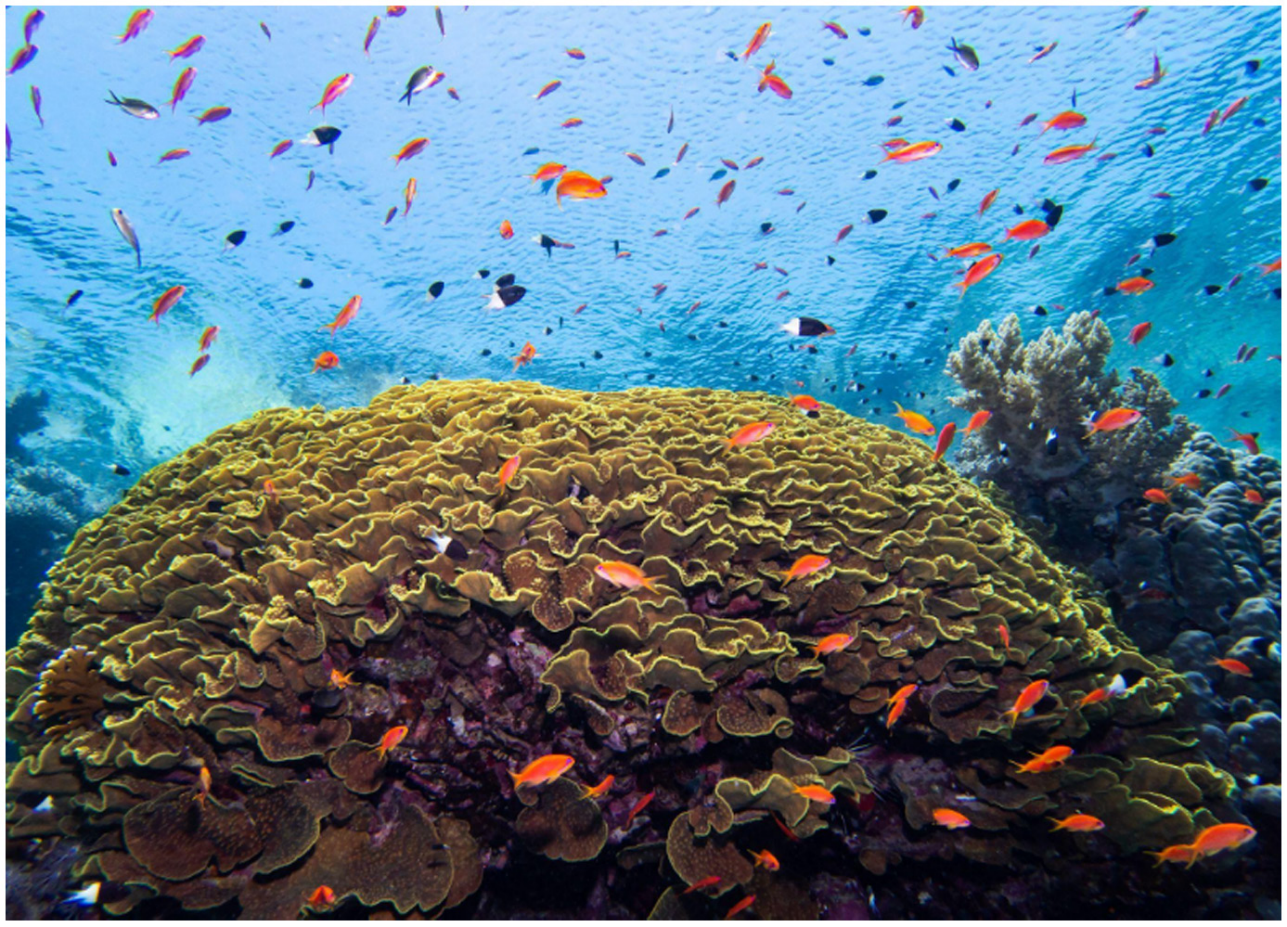
Coral reef 10‐m depth, Eilat, Red Sea (Credit; Shachaf Ben Ezra).

Reef building corals are highly photosensitive, and it is already clear that ALAN is a major emerging sensory pollutant concern for shallow coral reef ecosystems. Yet, ALAN is one of the most understudied threats to corals (Figure 6).

Tropical coral reefs are one of the most biodiverse and productive ecosystems on Earth. Their complex framework offers a unique habitat for thousands of associated species, supporting more species per unit area than any other marine ecosystem. In addition, coral reefs also provide important ecosystem services to millions of people (Hughes et al., 2017, 2018); yet, they have been heavily deteriorated worldwide due to poorly managed anthropogenic activities, habitat loss, and climate change (Hughes et al., 2018).

ALAN has been detected in fringing reefs localized in strongly urbanized locations. Mean night sky brightness levels at reef locations (see Ayalon, Rosenberg, et al., 2021) show that many coral reef areas worldwide are affected by ALAN. An applicable example is the coast in the Gulf of Eilat/Aqaba in the Red Sea, where most of the studies on the effects of ALAN on reef building corals have been conducted (e.g., Ayalon et al., 2019; Levy et al., 2020; Tamir et al., 2017). This region is heavily light polluted, and the light reflected from the cities surrounding the reef can be seen from space (Tamir et al., 2017).

Scleractinian corals in symbiosis with dinoflagellates (Symbiodiniaceae) are the foundation species for the formation of shallow water tropical coral reefs. Because their endosymbiotic partners perform photosynthesis—and up to 95% of the photosynthates can be translocated to the coral host for its metabolic needs—this symbiosis is at the basis of the success of such tropical environments (LaJeunesse et al., 2018; Muscatine et al., 1981; Trembley et al., 2012). The aspects of such symbiosis contribute to a higher susceptibility to ALAN, since corals and their symbionts are highly photosensitive and are mostly found in shallow, clear water with relatively high light levels (Rosenberg et al., 2019).

Light is detected by corals through light‐sensing molecules, such as cryptochromes (CRY)‐proteins that can convert light leading to changes in intracellular levels of important second messengers (Levy et al., 2007). The light/dark cycle regulates many cellular processes, the dark period being crucial for stress recovery and repair, especially for the photosynthetic symbionts (Hill et al., 2011; Levy et al., 2020). Natural periodic illumination (both solar and lunar) is a critical factor in cueing important processes of coral reproduction (Ayalon, Rosenberg, et al., 2021; Lin et al., 2021). In addition, the blue light spectrum—for example, present in LED lights—play a key role in coral growth, symbiont density, Chlorophyll *a* content, and photosynthetic rates (D'Angelo et al., 2008; Wijgerde et al., 2014). Therefore, it is not surprising that the exposure of corals to ALAN has been shown to have detrimental effects on coral/symbionts metabolism and reproduction (see below). Yet, ALAN is one of the most understudied threats to corals.

Molecular evidence in corals on the effects of ALAN generally match those of more complex organisms, mainly mammalians. Rosenberg et al. (2019) used transcriptomic analysis to compare corals of the species *Acropora eurystoma* growing under natural light cycles and under ALAN (50–40 lux). Authors demonstrated many pathways that were altered, with approximately 25 times more differentially expressed genes that regulate cell cycle, cell proliferation, cell growth, and protein synthesis under ALAN.

Physiological and biochemical investigations on Red Sea corals reported significant deleterious effects of ALAN. Ayalon et al. (2019) first demonstrated that *Acropora eurystoma* and *Pocillopora damicornis* experienced oxidative stress and photosynthetic impairment after exposure to different wavelengths of ALAN (40‐35 lux). Subsequently, a more detailed study by Levy et al. (2020) showed that the extent of deleterious effects of ALAN (40‐30 lux) on the symbiotic association (loss of symbionts and Chlorophyll content) and physiology of *Turbinaria reniformis* and *Stylophora pistillata* was aligned with the severity of the oxidative stress condition experienced by the species. The same study also presented preliminary evidence that corals presenting higher basal levels of antioxidant capacity, such as *Turbinaria reniformis*, may be more resistant to ALAN. Ayalon, Benichou, et al. (2021) also reported ALAN can influence Symbiodinaceae cultures and demonstrated different physiological responses according to the algae type. More specifically, *Clodocopium* type showed to be generally more sensitive compared to *Durusdinium* type, presenting decreases in the maximum quantum efficiency of PSII, in the mitotic index, and in total chlorophyll content after exposure to ALAN with illumination level up to 5 lux.

Regarding the effects on reproduction, recent studies reported large effects of ALAN on the gametogenesis and the synchronization of gamete release in prominent coral species from the Indo‐Pacific Ocean. The gametogenesis cycle of *Acropora millepora* and *Acropora digitofera* was delayed or masked by exposure to ALAN (~15 lux), leading to unsynchronized gamete release (Ayalon, Rosenberg, et al., 2021). Dim light during the night also suppressed spawning in coral *Dipsastrea specisosa* (Lin et al., 2021). Importantly, this later study showed that the period of darkness between sunset and moonrise is essential to trigger synchronized mass spawning. Additionally, Tamir et al. (2020) reported a 30% decrease in coral settlement success due to ALAN (~20 lux). Such results are alarming for the future of coral reefs. More than 80% of Scleractinian corals are broadcasting spawners (Baird et al., 2009), and asynchronous reproduction caused by ecological speciation could lead to reproductive isolation and prevent gene flow between differential lit coral communities (Rosenberg et al., 2019). Further, successful gamete production and fertilization, development of viable offspring, and survival of coral recruits are undoubtedly some of the most relevant processes for replenishing degraded reefs (Ayalon, Rosenberg, et al., 2021; Harii et al., 2010).

In addition to the symbiosis established with endosymbiotic dinoflagellates, corals are associated with prokaryotic symbionts. In fact, the coral host, and their microbiome (microalgal and prokaryotic symbionts) show a tightly intertwined metabolic activity (Thompson et al., 2015). Coral‐associated prokaryotic microbes are taxonomically and functionally diverse and are key for maintaining the health of the holobiont (Hernandez‐Agreda et al., 2018; Krediet et al., 2013; Olson et al., 2009). To date, only one study explored the effects of ALAN on the coral microbiome. Baquiran et al. (2020) showed that the overall microbial community structure of the coral *Acropora digitifera* remained stable under ALAN (~15 lux). However, it is important to note that bacteria that could use light for energy production (chlorophototrophic members of the phylum Proteobacteria), as well as those that are associated with the phototrophic symbionts of the coral, increased in abundance under light pollution conditions. Possibly, the higher abundance of symbiont‐associated microbes is linked to greater abundance and activity of the dinoflagellate symbionts under short‐term exposure to ALAN (Baquiran et al., 2020).

As for other species inhabiting coral reefs, only effects on fishes have been investigated so far. Fobert et al. (2019, 2020) showed a negative impact of ALAN (~15 lux) on the reproductive success of the common clownfish *Amphiprion ocellaris*, with an increased interval between spawning events and a smaller size of eggs in comparison to dark conditions; in addition, hatching success was affected both by the presence of ALAN and by its spectral composition, with a more negative effect of cool‐white in comparison to warm‐white light. After hatching, other life stages might represent critical intervals for the persistence of fish populations under light pollution. O'Connor et al. (2019) highlighted larvae of *Acanthurus triostegus* living 10 days post‐settlement under ALAN conditions (~20–25 lux) experienced higher mortality rates by the end of the experiment, although growing faster and heavier than control ones. Furthermore, a long‐term study conducted in the wild (Schligler et al., 2021) showed that environmentally relevant intensity of ALAN (~4.3 lux) is also able to reduce survival and growth of juveniles of the anemonefish *Amphiprion chrysopterus*), compared with individuals exposed to natural night illuminance (by moonlight). Finally, a recent study on the blue green *Chromis viridis* highlighted complex sub‐acute effects of ALAN on adult fish, with predator threat able to alter the increased metabolism of both specific tissues and whole organism observed under ALAN conditions (~100 lux). Although an evolving area of study, it is already clear that ALAN is a major emerging sensory pollutant of concern for shallow coral reef ecosystems. ALAN acts as a chronic disturbance, and corals under such pressure may not be able to perform their normal cyclic behaviors (Rosenberg et al., 2019). Therefore, ALAN may impact the future of coral reefs, eventually contributing to global coral reef decline.

### 2.4 Pelagic environment organisms

**FIGURE 7:**
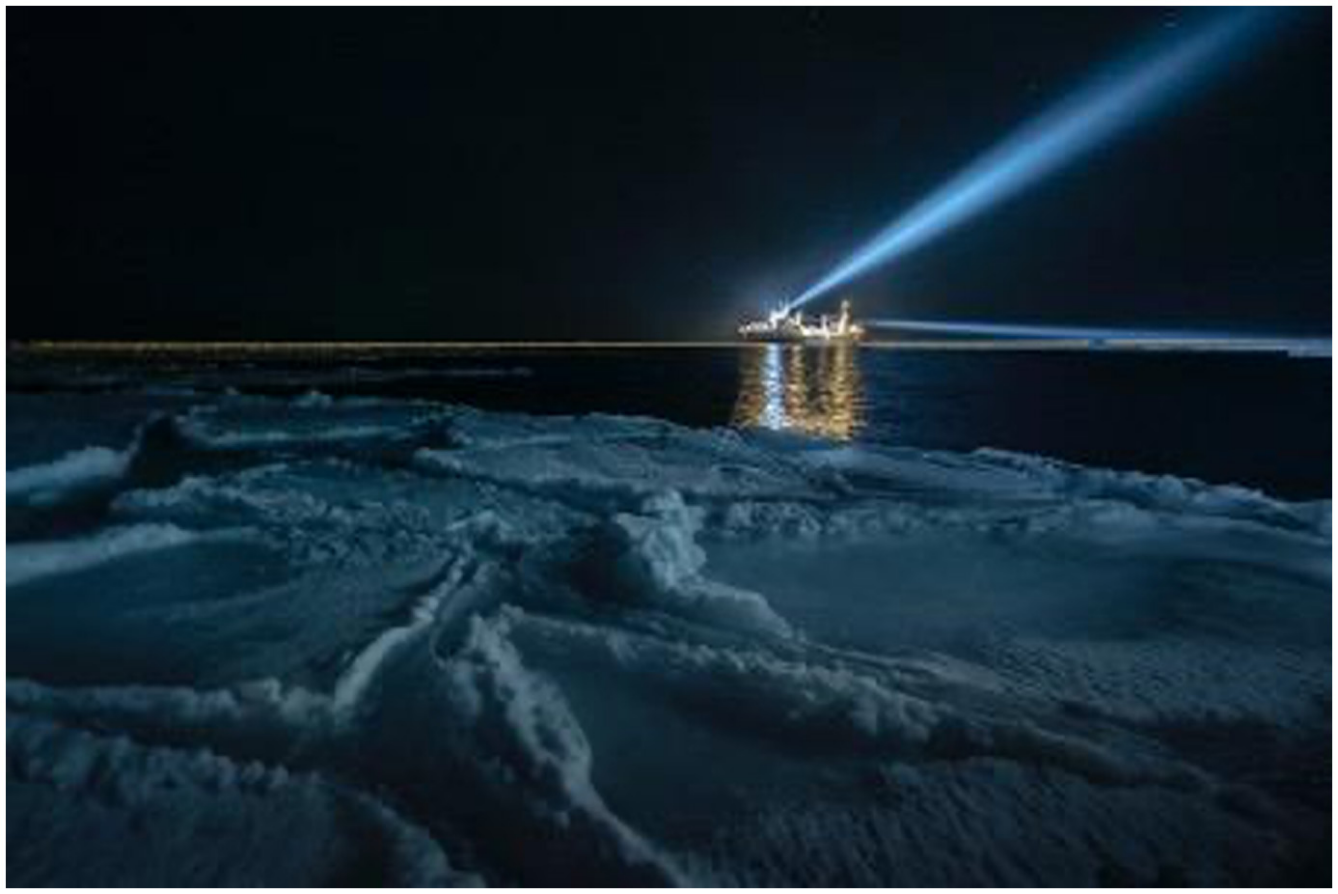
Lights from a ship working in the dark (Credit; Mike Snyder).

Oceans are vast, three‐dimensional, and mostly influenced by currents. Pelagic organisms are not attached to a substrate, hence the direct effects from ALAN in the open oceans are likely to be different from those on a beach or on the seafloor. Still, recent work suggests that lights from ships may have an impact on organisms in both the epipelagic and even mesopelagic zones (Figure 7).

Light influences pelagic organisms in many ways, and artificial light may have a strong impact in their behavior (Blaxter & Currie, 1967). Prey availability, limiting the initiation and magnitude of phytoplankton blooms and mortality through visual predation are some examples of how artificial light may have an impact. In general, most zooplankton are negatively phototactic (Forward Jr, 1988), migrating to depth during daylight to avoid the threat of visual predation, and surfacing at night to feed. This behavior is called Diel Vertical Migration (DVM) (Brierley, 2014). DVM is a characteristic feature of all the world's oceans and is considered the largest synchronized movement of biomass on the planet (Hayes, 2003). It is thus an important factor in structuring pelagic communities. At the same time, as this process is mediated by light (Ringelberg, 2010), it may potentially also be strongly affected by artificial light (Berge et al., 2020). At latitudes characterized by midnight sun during summer and polar night during winter, DVM was generally assumed not to occur except during spring and autumn periods when there is a clear day‐night cycle (Berge et al., 2009). This view, however, was recently challenged. Instead of an ecosystem that enters a resting state during the polar night, we now recognize a highly active ecosystem characterized by continuous activity and biological interactions across all trophic levels and taxonomic groups (Berge et al., 2015). Importantly, even at the darkest periods of the year, light is still the primary regulative factor for most of these interactions, including vertically migrating zooplankton (Last et al., 2016; Ludvigsen et al., 2018).

It is important to note that behavioral responses to artificial light vary among taxa. While some species are known to be attracted to light, with herring *Clupea harengus* (Stickney, 1969), krill (Krafft & Krag, 2021; Utne‐Palm et al., 2018), snow crab *Chionoecetes opilio* (Nguyen et al., 2020), and grey mullet *Mugil cephalus* (Marchesan et al., 2005) as well‐known examples, others are known to avoid light. North Atlantic and Arctic copepods (Ludvigsen et al., 2018), Atlantic cod *Gadus morhua* (Utne‐Palm et al., 2018), and sea bream *Sparus auratus* (Marchesan et al., 2005), all species that are commercially important, have been shown to avoid ALAN. A study from the Red Sea, in which a ROV equipped with LED lamps were used to herd mesopelagic scattering layers, similarly concluded that artificial light attracted some species and repelled others (Kaartvedt et al., 2019). Also, recent studies from the high Arctic Archipelago of Svalbard have shown that artificial light from both ships and instrumentation may have a strong impact on organisms down to at least 200 m depth (Berge et al., 2020). The artificial lights in this case were measured to 2.2 μmol photons m^−2^ s^−1^ at the sea surface. However, with field experiments carried out across nearly 8 degrees of latitude, differences in response to artificial light varied both qualitatively and quantitatively in a way that could not be explained by species composition alone. Hence, in addition to interspecific differences in responses to light (Ryer et al., 2009), intraspecific variation could also complicate interpretations of responses to artificial light (Berge et al., 2020).

Effect of light pollution and ALAN in the open ocean is difficult to assess. By default, sampling in the open ocean is often biased, as organisms are not attached or restricted to a specific “site” or physical habitat. And except for acoustic instruments, most sampling technologies include the use of either artificial light or the instrument itself creates a shadow that might influence the organisms (see Box 2). To examine the potential effect of artificial light is, thus, very difficult to do with traditional methodologies that use artificial light to function. A significant “effect” of ALAN in the open ocean is therefore not restricted to direct effects but will also to a large degree include accuracies and artifacts in the measurements itself.

BOX 2 **How research activities can interfere and create bias due to artificial light**

Despite a growing body of literature reporting behavioral disturbance of marine organisms exposed to artificial light, external light sources remain widely used in oceanography and marine ecology studies. Advances in optical technology, combined with the increased desire to use non‐lethal observation approaches, have driven the development of new sensors and instruments to document marine ecosystems (Bicknell et al., 2016), but these instruments generally require an external light source. For example, Optical probes such as the Underwater Vision Profiler (Picheral et al., 2010), the Laser‐Optical Plankton Counter (Basedow et al., 2013; Herman & Harvey, 2006), the Video Plankton Recorder (Sainmont et al., 2014), and the Light frame On‐sight Key species Investigation system (Schmid et al., 2016; Schulz et al., 2010) all use light sources and optical sensors to assess the vertical distribution and abundance of zooplankton. Researchers and the industry alike increasingly use high‐definition (HD) video cameras or stereo‐cameras mounted on trawls to document the catchability of different species or size classes of fish (Boldt et al., 2018; Underwood et al., 2020; Williams et al., 2010). Such camera systems, when used in dim environments, rely on external light sources to distinguish, and identify marine animals at depth. Although previous studies have raised concerns about the impact of artificial visible light on measurements from optical instruments (Benoit‐Bird et al., 2010; Boldt et al., 2018; Doya et al., 2014; Trenkel et al., 2004; Widder et al., 2005), these biases have rarely been quantified (Bicknell et al., 2016). Nonetheless, artificial lighting is assumed to be the main source of biases in fish surveys using cameras and underwater vehicles (Rooper et al., 2015; Ryer et al., 2009; Stoner et al., 2008). The use of red light has been suggested for marine surveys requiring external light sources because it is assumed that most species do not react as much to red light as to shorter wavelengths, such as blue or green (Boldt et al., 2018; Rooper et al., 2015; Widder et al., 2005). In support of this hypothesis, Peña et al. (2020) and Underwood et al. (2020) deployed oceanographic probes equipped with different light colors and showed that mesopelagic (200–1000 m) fish avoid white, blue and green, but not red light. This, however, was recently challenged by Geoffroy et al. (2021) who, using hull‐mounted echosounders above an acoustic probe or a baited video camera, each equipped with light sources of different colors (white, blue, and red), demonstrated that pelagic organisms in Arctic and temperate regions strongly avoid artificial light, including visible red light (575–700 nm), from instruments lowered in the water column. Light levels varied within the range of 11–18 μW cm^−2^ for the colors tested (see Geoffroy et al., 2021 for details). The density of organisms decreased by up to 99% when exposed to artificial light and the distance of avoidance varied from 23 to 94 m from the light source, depending on colors, irradiance levels and, possibly, species communities (Geoffroy et al., 2021).

### 2.5 Seabirds

**FIGURE 8:**
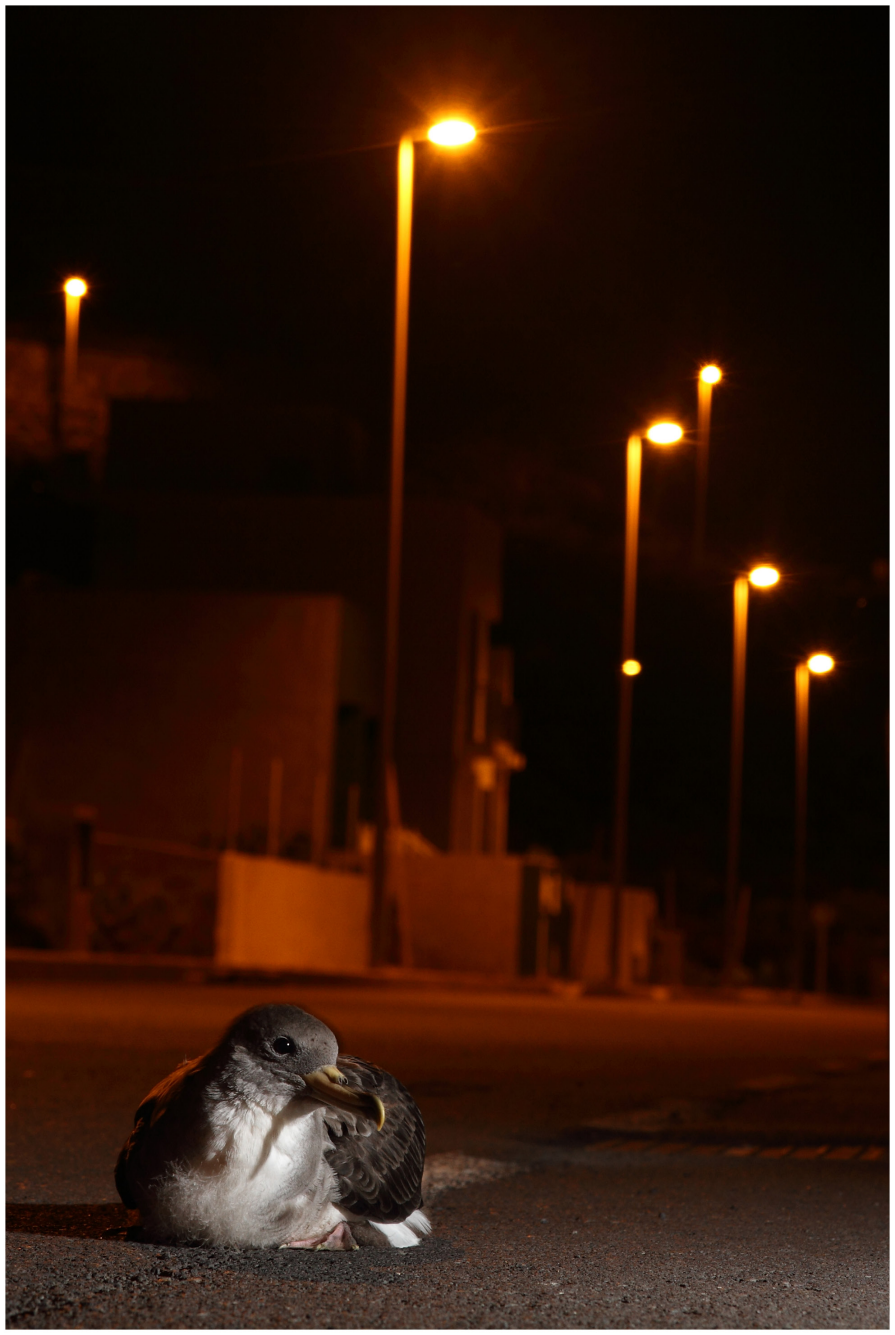
Cory's shearwater *Calonectris borealis* fledgling grounded by artificial light in Tenerife, Canary Islands (Credit; Beneharo Rodríguez).

Light pollution causes massive mortality events on seabird fledglings, involving more than 70 species, some of them severely threatened. More subtle effects of ALAN are still poorly understood (Figure 8).

Seabirds are defined as avian species for which a large proportion of the population relies on the marine environment for at least part of the year (Croxall et al., 2012). For example, petrels spend most of their life at sea and only visit land for breeding (Brooke, 2004), while some gull species can spend most of their lives outside the ocean. Within marine biota, seabirds are the only animals mastering the three environments: marine, terrestrial, and aerial. Thus, there is a trade‐off among the adaptations of seabirds to cope with different environments. Seabirds are one of the most threatened groups of birds (Dias et al., 2019). According to the International Union for Conservation of Nature (IUCN) Red List criteria, around 31% of all seabird species are globally threatened and 47% have declining population trends.

Environmental conditions, such as light, can rapidly change. Many seabird species nest underground or visit their breeding colonies at night while foraging during daylight. In addition, many seabirds forage by diving in the water column, where the light spectrum changes rapidly with depth (Regular et al., 2011). Seabirds must adapt to rapid changes in intensity and spectra of light to survive. In fact, visual systems of diving seabirds are more sensitive to blue light, the light that penetrates more in‐depth (Martin, 2017). The increase of artificial light levels has been identified as a threat for seabirds and, particularly, petrels (Dias et al., 2019; Rodríguez et al., 2019).

Seabirds encounter the most light‐polluted areas on land, mostly coastal areas close to or within their nesting colonies. These light sources, such as streetlights, road lights and lighthouses, attract and disorient birds during their flights between colony and foraging sites at sea. Many seabirds visit colonies at night presumably to avoid predation by diurnal predators (Bourgeois et al., 2008; Rubolini et al., 2015). Visit frequency of breeders is influenced by moon cycles probably because of moonlight level variation (Riou & Hamer, 2008; Rodríguez et al., 2016). Thus, breeders could be deterred from visiting colonies when lights are turned on close to their nests or colonies. A recent experimental study on breeders of Manx shearwater *Puffinus puffinus* demonstrated that adult breeders flying over the colony were deterred by blue and green light in comparison to red light (Syposz et al., 2021). In addition, the number of birds flying over the colony decreased with the duration and intensity of light treatments (Syposz et al., 2021). However, experimental studies on the effect of color and intensity of light (3, 15, and 100 lux) on the colony attendance of the smallest and only penguin whose activity on land is strictly nocturnal, the little penguin *Eudyptula minor*, demonstrated that penguins preferred lit paths over dark paths to reach their nests (Rodríguez et al., 2018).

From a conservation point of view and in a short‐scale term, the most negative consequence of light pollution is direct mortality caused mainly on fledglings of underground‐nesting seabird species (Rodríguez, Holmes, et al., 2017). This phenomenon is known as fallout, and it occurs in all the oceans and seas across the world. Although reasons are unknown (Atchoi et al., 2020), fledglings of many petrel species, but also Alcids and some sea ducks, are attracted and/or disorientated by lights during their maiden flights from their nests toward the ocean (Rodríguez, Holmes, et al., 2017). This leads to grounding and hitting infrastructures, for example, wires, antennas, buildings, or even the ground, causing injuries and fatal victims. If they survive the first collisions, they are vulnerable to other threats, such as vehicle collisions, predation by domestic animals, or traps where they die of inanition or dehydration because they are usually unable to take off. Rescue programs are initiatives aiming to mitigate light‐induced mortality. To reach this, they call for the public implication in rescuing and reporting birds grounded at lit areas, main towns, and cities. Most birds admitted to rescue programs survive (~ 90%), but the fraction of grounded birds that never are found or reported through these collaborative initiatives is unknown. Some studies indicate that around 40% of birds are never rescued (Ainley et al., 2001; Rodríguez et al., 2014). Artificial light could also affect the natural colony attendance of breeders visiting colonies at night.

Increasing ALAN levels in and around nesting colonies could impact seabird's breeding behavior and, consequently, chick provisioning. In an overnight weight gain study, Scopoli's shearwater *Calonectris diomedea* chicks situated closer to a high‐light intensity disturbance (i.e., disco event) gained less weight compared to conspecifics from nests further away (Cianchetti‐Benedetti et al., 2018). Such effects were not perceivable at fledging, but it is expected that a higher frequency of disturbance events could affect chicks' fitness and, even, breeding success.

Beyond the coastline, seabirds can also encounter extremely light‐polluted areas associated to offshore oil and gas platforms, vessels, or light‐enhanced fisheries, for example. Our knowledge on light‐induced mortality at sea is quite limited (Gjerdrum et al., 2021; Glass & Ryan, 2013; Merkel & Johansen, 2011; Ronconi et al., 2015; Ryan et al., 2021), although we know that adults can also be involved in attraction episodes. Clear monitoring protocols and independent trained observers, who could rely on technological advances, such as telemetry, thermal cameras, acoustic recordings, and radar, are essential to record episodic seabird‐light interactions at sea (Ronconi et al., 2015).

The increase of light pollution levels under water (see above) widens the photic zone at night, but also during dawn and dusk. Thus, both at neritic and oceanic waters the increase of light levels by ALAN could enhance the foraging of pursuit‐diving visual predator seabirds, such as murres and penguins (Cannell & Cullen, 1998; Elliott & Gaston, 2015; Regular et al., 2011).

Artificial lights can also concentrate prey, which seabirds then take advantage of. Several gull species have been reported to increase their foraging opportunities on marine, coastal and terrestrial lit areas. For example, fishing vessels usually use light to concentrate fish and squid. In the Mediterranean Sea, lights of fishing vessels favor the capture of fish by the Audouin's gull *Ichthyaetus audouinii* by illuminating the sea surface, concentrating fish, and locating shoals (Arcos & Oro, 2002). Similarly, Brown‐hooded gulls *Larus maculipennis* predate on Isopoda, Polychaeta, fish larvae, and crustaceans, which are concentrated under artificial lights on Argentinian coastal piers (Leopold et al., 2010). On land, Black‐backed gull *Larus dominicanus* can take advantage of Cerambicidae beetles attracted to artificial lights at sawmills (Pugh & Pawson, 2016). Gulls can also prey on seabirds and such predation can be facilitated by artificial light. At Benidorm Island (western Mediterranean), Yellow‐legged gulls *Larus michahellis* increased predation on European storm‐petrels *Hydrobates pelagicus* after light levels increased by a new light installation in the nearby Benidorm city (Oro et al., 2005).

Although there is a certain consensus about the higher pervasiveness of blue light for wildlife and, particularly, seabirds (Longcore et al., 2018; Rodríguez, Dann, & Chiaradia, 2017; Syposz et al., 2021), more research is needed on the spectrum of light in the perception of seabirds. Similarly, reductions in the duration of lights, by means of smart‐lighting or part‐night lighting, could help to mitigate light pollution impacts, but current understanding is insufficient to underpin sound recommendations for all species. For example, more research is needed to assess the threshold levels from which a response is triggered as well as the relative intensity with ambient light levels (e.g., during full and new moon nights). The distance from light sources and seabirds at which they are attracted or disorientated must be influenced by light intensity. Thus, determining distances at which individuals are safe is crucial for managing breeding colonies and corridors between colonies and the ocean for inland breeding species. GPS tracking has revealed that most of Cory's shearwater *Calonectris borealis* fledglings are grounded on areas distant <16 km from their nests (Rodríguez et al., 2015, 2022).

## 3 CONSERVATION GUIDELINES AND STRATEGIES

Some few countries today—Spain (Catalonia), Chile, France, and Italy (Piedmont)—are trying to establish laws to regulate light pollution. Most of the applicable documents addressing ALAN are guidelines, Codes or Standards issued by regulators, advisory committees, non‐government organizations (NGO) or professional bodies with no legal basis for enforcement of recommendations. Many of the professional body guidance documents are targeted at lighting engineers or designers, provide little detail regarding ALAN management and mitigation for the protection of sensitive receptors and must be purchased at a substantial cost (e.g., AS/NZS 4282, 2019). In addition, regulators, advisory committees, and NGOs typically focus on a single sensitive receptor such as bats, birds or dark sky conservation for astronomy or star gazing (City of Calgary, 2011; NSW, 2016; Voigt et al., 2018).

Following the adoption of the Australian National Light Pollution Guidelines for Wildlife, including Marine Turtles, Seabirds and Migratory Shorebirds (Commonwealth, 2020; CMS, 2020a, 2020b) by the UNEP Convention of Migratory Species in 2020, the CMS Secretariat has expanded on the issue with the release of a follow‐up review of ALAN impacts on migratory species not covered by the Australian guidelines (CMS, 2021). This document summarized some of the available international laws and guidelines that address ALAN. Except for the CMS guidelines (CMS, 2020b), laws, standards and codes all relate to terrestrial based ALAN and sensitive receptors. Guidance for addressing the impacts and management of artificial light in the marine environment does not currently exist in the (English) grey literature or publications. Recognizing the difficulties in setting specific assessment trigger values for the broad range of variables influencing the impact of light on wildlife, the CMS guidelines recommend a case‐by‐case approach to ALAN impact assessment, management, and mitigation. The conservation strategies adopted will vary depending on the sensitive receptor (foraging and migrating birds, hatchling sea turtles migrating offshore, plankton, fish, marine mammals etc.), the sensitivity of the receptor to different wavelengths of light (e.g., whales do not see color), the light sources (ships at anchor offshore, offshore oil and gas facilities and flaring, ports and marinas, slow moving dredge vessels etc.), the light features (wavelength/color, intensity, shielding, flaring gas flow rates, elevation) and variables such as turbidity, water depth, clouds, dust, aerosols, moon phase, fog, day length, all acting in combination to influence the visibility of the light. Standard best practice guidelines for outdoor lighting design for the protection of sensitive receptors including wildlife have been published (ADSA, 2021; CMS, 2020b; IDA, 2021) and include: (i) Start with natural darkness and only add light for specific purposes; (ii) use adaptive light controls to manage light timing, intensity, and color; (iii) light only the object or area intended, (iv) use the lowest intensity lighting appropriate for the task; (v) use non‐reflective, dark‐colored surfaces; and (vi) use lights with reduced or filtered blue, violet, and ultraviolet wavelengths. Of these, the most important is avoiding short wavelength blue light due to its ubiquitous visibility across a wide range of taxa, as well as its higher capacity to penetrate the water column, shielding light to prevent light spill into the water or sky, and minimizing light intensity.

**FIGURE 9:**
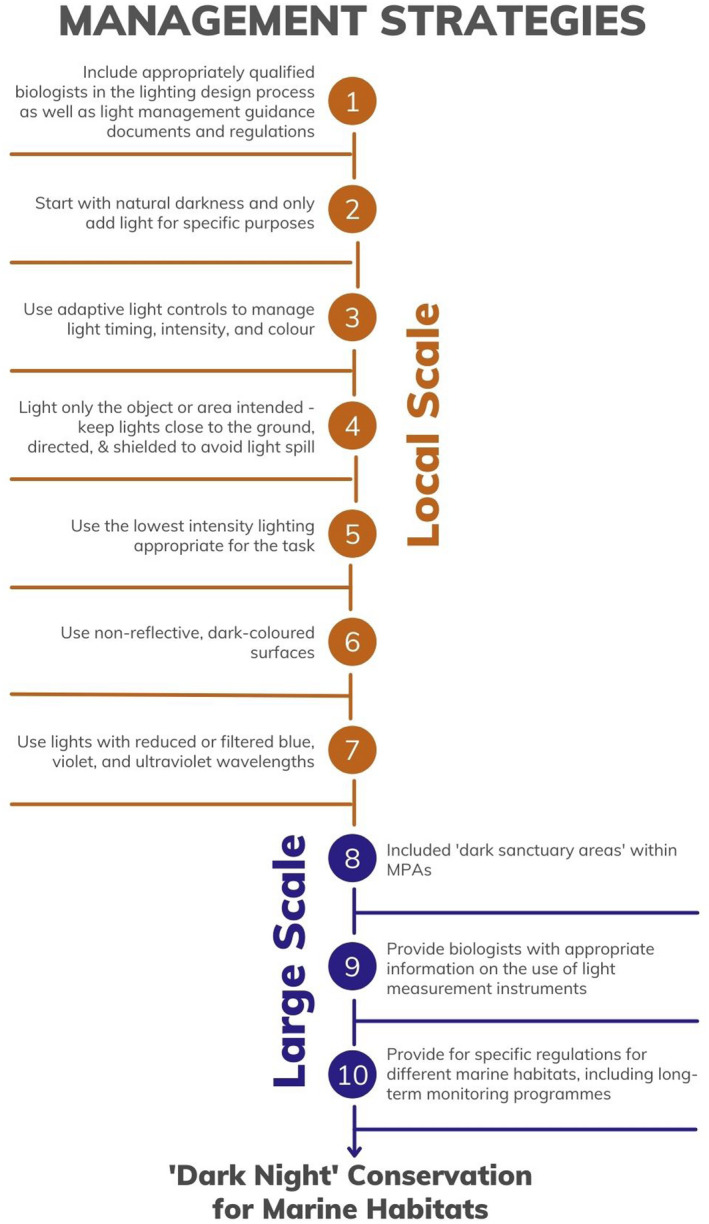
Ten golden rules for dark night conservation for marine habitats.

A correct application of such rules, for the management of “dark nights” in the marine environment, needs the consultation of appropriately qualified biologists, which should be included in the lighting design process as well as light management guidance documents and regulations. At a larger scale, qualified researchers should help identify appropriate “dark sanctuary areas” within MPAs and providing for specific regulations in different marine habitats, including long‐term monitoring programs. Considering the best practices guidelines and their correct application, we propose here “Ten golden rules for dark night conservation for marine habitats” (See Figure 9).

## 4 CONCLUSION AND PERSPECTIVES

Progress in our understanding of ALAN impacts in marine ecosystems has accelerated dramatically over the last 5 years. The number of species, habitats and ecological processes with documented responses now present a compelling case for ALAN as a globally widespread pollutant that is reshaping nature across our coastlines. The field remains however, recently emergent, and numerous knowledge gaps exist that if addressed would aid in the prediction and mitigation of ALAN impacts in the sea. Here, we highlight key questions for future research to be addressed:

1. What is the impact of artificial light at sea on marine wildlife populations?
2. What is the contribution of marine traffic (i.e., mobile light sources from vessels) and traffic management (i.e., fixed light sources from navigation markers) to marine light pollution, and what is their potential to impact marine biodiversity?
3. What are the indirect impacts of ALAN in marine ecosystems through species interactions and trophic cascades?
4. Are there intergenerational impacts of ALAN?
5. What are the best practice techniques to monitor and measure biologically meaningful (i.e., radiometric as opposed to photometric) light, both underwater and on land, at both fine (meters) and broad (kilometers) scales?
6. What are the thresholds (intensity, wavelength, exposure time) that elicit biological responses in marine species?
7. How does the disruption of moonlight cycles by artificial skyglow impact circalunar rhythms in marine organisms?
8. What is the impact of coastal ALAN on marine ecosystem services?
9. Does ALAN impact long distance mass migrations of marine megafauna?
10. Is there temporal variability (e.g., monthly, or seasonal) in ALAN impacts on marine ecosystems?
11. Are there additive/interactive effects between ALAN and other anthropogenic disturbances?

Addressing these questions will provide insight into the full extent of ALAN impacts in marine ecosystems, their consequences for society, and options for mitigating them.

## CONFLICT OF INTEREST

Authors declare that they have no competing interests.

## Data Availability

Data sharing is not applicable to this article as no new data were created or analyzed in this study.
